# Prof. Dudley’s Surgical Papers

**Published:** 1849-11

**Authors:** 


### PROFESSOR DUDLEY'S SURGICAL PAPERS.

We were pleased, some time since to see that a medical journal was to be published in Lexington, Ky., and that among its claims to the confidence and support of the profession, was an announcement that Professor B. W. Dudley would conttibute to its pages the results of his long and useful career as a surgeon. The eminent opportunities he has enjoyed in practice, for contributing to the advancement of surgical science, made us hail this announcement with no ordinary pleasure. We know of no American surgeon who has had it in his power to do more for his art, and we have often regretted that he has done so little of a permanent character. We are not, by any means, fond of the mere book-maker, but Prof. Dudley owed to his profession much more extensive printed contributions than he has made. Sir Astley Cooper, in the midst of labors as a teacher and practitioner of surgery, quite as onerous as those of Professor Dudley, found time to publish a series of works on surgery that will live as long as surgery is practiced as an art, and which constitutes an imperishable monument to his fame. The motto of these works is that which every man who has had Prof. Dudley's opportunities should wear as a frontlet to the eyes—"Duty recognized, and duty performed." We should rejoice to be able to say that Prof. Dudley has done in this respect, what the profession had a right to expect from him. He was the founder of western surgery, and for a long series of years, he justly enjoyed a monopoly of all the important surgery of the Ohio and Mississippi vallies. Of his eminent abilities as a surgeon, there never was a doubt, and his means of observation have been superior to those of any other American surgeon, Dr. Physic alone excepted. He has also long been eminent as a surgical teacher, and with his various and ample opportunities of usefulness, we repeat, his professional brethren had a right to expect from him some contributions, of a permanent character, to surgical science. Hitherto these just expectations have not been realized. Years have been permitted to rush by as the winds, and no advantage was taken of their gliding hours. We fear that it is now too late for Professor Dudley to do much as a writer, that will be equal to his reputation, or that will have the effect of exalting his favorite department of medicine. Author-craft comes not in the evening of life, at the call of any one, or from the mere desire for it.—As in all other matters of human excellence, it requires discipline, training and experience, and these were all neglected, by Prof. Dudley, up to a period when we fear they can be used to little or no advantage. It is with more than ordinary regret that we indite these sentiments, and we should greatly prefer, if the opportunity were afforded to us, to say that Professor Dudley had answered the just expectations of his medical friends, by a work on surgery worthy of his great reputation. But the golden opportunity for such a work has been permitted to trend its way into the grave of past years, and it cannot be wooed back, even by more assiduous courtship than Prof. Dudley is likely to give it.

One of our collaborateurs in the present number of the Journal, has reviewed Prof. Dudley's first contribution to the Lexington Medical Journal, in a style somewhat more severe than we should have employed, but the criticism is one of much ability, and has no spirit of unfairness. While commenting on Prof. Dudley as an author, the writer does justice to his merits as a surgeon, and no doubt feels regret that he could not say more creditable things of Dr. Dudley's authorship. The judgment of the profession will coincide with that of the reviewer, and with more of a feeling of sorrow than of anything else.

Professor Dudley has done much himself to defeat the success of his printed contributions to surgery. He was so constantly in the habit of hacking at "the books," in his lectures, that he created a distaste for any kind of reading, and made the élèves of his courses look with suspicion upon all printed principles. He always seemed to look upon surgical books as antagonistic to himself, and as enemies who were to be cuffed whenever an opportunity presented itself. The direct effect of these sneers at "the books" was to narrow and confine the minds of students, and to make them conclude that the horizon of surgical instruction was the walls of the amphitheater, beyond which sunrise was unknown.

We look back upon Professor Dudley's teaching with the warmest feeling of reverence, tempered with sorrow that he has been so unjust to his own great fame. The world would not willingly let the name of such a man as Professor Dudley die, except by his own act. Age has overtaken him entirely unprepared for the work, which he now shows he feels should be done; the duty is recognized, when it is too late to perform it. "Fast anchored in the past" stands all hope of success, and futile are all efforts to release it from the slough of time-worn neglect. None but those who have experience can know the trials and discipline necessary to successful composition. The painful study, the sleepless nights, the repeated revision, and constant siftings of thought are unknown to all but the initiated. One who knew of what he was speaking, uttered, in a witticism, a sound canon in rhetoric—it is: "Easy writing makes hard reading." We shall rejoice, however, to find in Professor Dudley's future papers on surgical practice, some of those redeeming traits, that are absent from his first. B.

